# Coating-free TiO_2_@β-SiC alveolar foams as a ready-to-use composite photocatalyst with tunable adsorption properties for water treatment†

**DOI:** 10.1039/c9ra09553e

**Published:** 2020-01-22

**Authors:** Marisa Rico-Santacruz, Patricia García-Muñoz, Clément Marchal, Nelly Batail, Charlotte Pham, Didier Robert, Nicolas Keller

**Affiliations:** Institut de Chimie et Procédés pour l'Energie, l'Environnement et la Santé (ICPEES), CNRS, University of Strasbourg 25 Rue Becquerel Strasbourg France garciamunoz@unistra.fr nkeller@unistra.fr; SICAT, SARL 20 Place des Halles 67000 Strasbourg France

## Abstract

Coating-free TiO_2_@β-SiC photocatalytic composite foams gathered within a ready-to-use shell/core alveolar medium the photocatalytically active TiO_2_ phase and the β-SiC foam structure were prepared *via* a multi-step shape memory synthesis (SMS) replica method. They were fabricated following a sequential two-step carburization approach, in which an external TiC skin was synthesized at the surface of a β-SiC skeleton foam obtained from a pre-shaped polyurethane foam during a first carburization step. The adsorption behaviour of the shell/core TiO_2_@β-SiC composite foams towards the Diuron pollutant in water was tuned by submitting the carbide foams to a final calcination treatment within the 550–700 °C temperature range. The controlled calcination step allowed (i) the selective oxidation of the TiC shell into a TiO_2_ crystallite shell owing to the β-SiC resistance to oxidation and (ii) the amount of residual unreacted carbon in the foams to be tuned. The lower the calcination temperature, the more pronounced the adsorption profiles of the composites and the higher the Diuron amount removed by adsorption on the residual unreacted carbon. The ready-to-use TiO_2_@β-SiC composite foams were active in the degradation of the Diuron pesticide, without any further post-synthesis immobilization or synthesis process at the foam surface. They displayed good reusability with test cycles and benefitted from an enhanced stability in terms of the titania release to water.

## Abstract 2

Coating-free TiO_2_@β-SiC photocatalytic composite foams gathering within a ready-to-use shell/core alveolar medium the TiO_2_ photocatalyst and the β-SiC foam structure were prepared *via* a multi-step shape memory synthesis (SMS) replica method.

## Graphical abstract

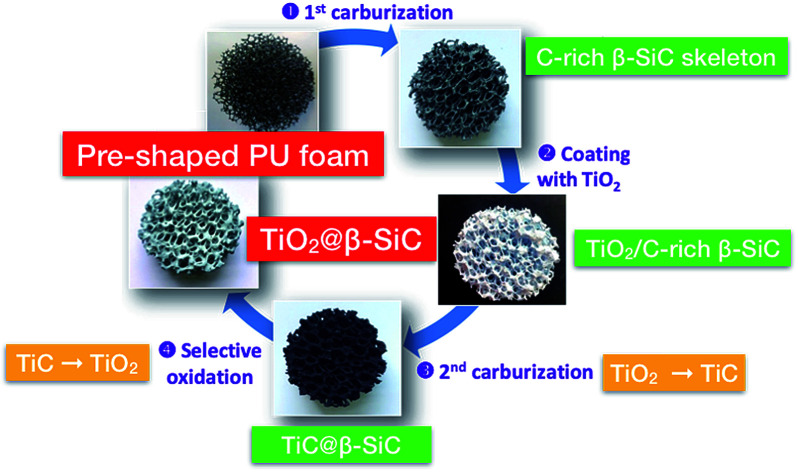

## Introduction

In the wastewater treatment, photocatalysis is one of the advanced oxidation processes that has demonstrated an ability to degrade and mineralize biorecalcitrant refractory compounds that cannot be eliminated by conventional treatments, or at least to degrade them into readily biodegradable compounds.^1,2^ Photocatalysis is usually carried out at the lab scale with nanoparticle suspensions, taking advantage of the maximum irradiated and exposed surface for optimizing the degradation activity. However, the necessary implementation of time-consuming and costly nanofiltration recovery steps for separating the powdery photocatalyst from the treated water, as well as safety issues related to its handling, make the process less viable for real applications and it also presents a less secure environment for recycling and handling.

Therefore, in parallel with the implementation of approaches based on the advanced design of nanomaterials for enhancing the activity of powdery photocatalysts,^3–6^ strategies dealing with the immobilization of photocatalysts – and more globally with the elaboration of macroscopic photocatalytic structures – were investigated for designing wastewater treatment processes that would allow the photocatalysts to operate in a continuous mode. In this vein, among cellular monolithic solids, metallic and ceramic open-cell alveolar solid foams have recently attracted significant interest for use as photocatalyst supports in water treatment to take advantage of a static mixer effect inside the reactor and due to their better light transmission than honeycomb- or square-channel monoliths.^7–11^ In addition, their open structure enables the photocatalyst to operate at an ultra-low pressure drop.^7^

Among ceramic foams, medium surface area self-bonded β-SiC foams have attracted strong interest thanks to their high thermal and chemical stability that allow them to be submitted to a wide range of severe conditions during the photocatalyst immobilization step, the reaction itself or thermal/chemical regeneration steps (if required). Further, the β-SiC foam surface is composed of a thin amorphous nanolayer exposing a high density of oxygenated surface groups, thereby favouring the anchorage of photocatalysts.^7–9,12^

Binderless β-SiC alveolar foams have been already used as a TiO_2_ support in water treatment.^7,12–14^ However, preparation of the foam-supported TiO_2_ photocatalysts requires immobilizing or synthesizing TiO_2_ onto the β-SiC support through a separate post-synthesis process, such that the TiO_2_/β-SiC foams can suffer detrimental stability problems that could provoke a release of the photocatalyst to water. The resistance of the catalyst–support interface towards the strains, derived from particle-to-particle and particle–fluid mechanical interactions in the reactor environment, remains indeed an essential quality criteria for a good support, especially, in order to avoid the release of photocatalyst particles from the support.

This work aimed at elaborating TiO_2_@β-SiC photocatalytic composite foams that gather the TiO_2_ photocatalyst and the β-SiC alveolar foam within a ready-to-use (coating-free) photocatalytic media, which consequently would not require the implementation of any post-synthesis immobilization or synthesis process at the foam surface. The foams were elaborated through a sequential multi-step carburization synthesis approach derived from the single carburization shape memory synthesis (SMS) replica method developed by SICAT catalyst for synthesizing pure self-bonded porous β-SiC foams from a pre-shaped polyurethane foam.^15,16^ Further, we aimed to control the adsorption properties of the ready-to-use TiO_2_@β-SiC composite foams towards the pollutant in water.

Diuron (C_9_H_10_Cl_2_N_2_O, 3-(3,4-dichlorophenyl)-1,1-dimethylurea), one of the most used contact herbicides from the family of substituted phenylureas, was taken as a model substrate to degrade for evaluating the liquid-phase photocatalytic activity of the TiO_2_@β-SiC composite foams under simulated solar light. Diuron is known to be degraded by photocatalysis *via* a well-accepted multi-pathway degradation route, involving mainly hydroxyl OH˙ radicals as active species,^17–19^ and consisting first in the attack of the alkyl function or in the chlorine substitution, followed by hydroxylation steps leading to the formation of acetic, formic and oxalic acid as the last short-chain acid reaction intermediates.^1,15,20,21^

## Experimental part

### Synthesis of the TiO_2_@β-SiC photocatalytic composite foams

**Fig. 1:**
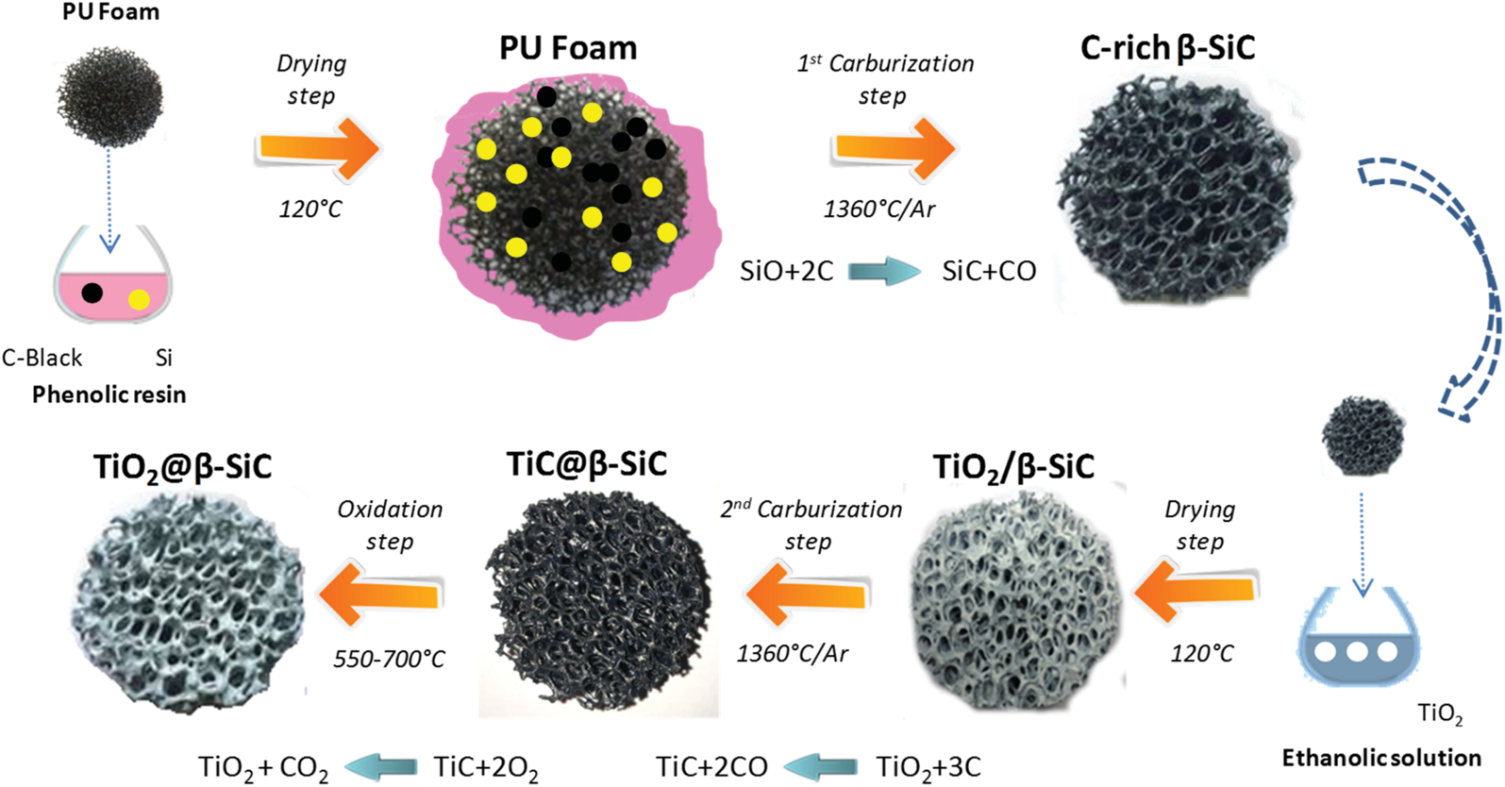
Schematic diagram of the sequential two-step carburization SMS replica synthesis of the shell/core TiO_2_@β-SiC composite foams.

Alveolar shell/core TiO_2_@β-SiC composite foams with medium surface area were jointly developed at ICPEES and at the SICAT company (Strasbourg, France) according to a sequential multi-step carburization SMS method, adapted from the single carburization SMS method owned by SICAT for synthesizing self-bonded porous β-SiC foams from a pre-shaped polyurethane foam.^22^ This two-step carburization SMS replica approach, schematized in Fig. 1, implements a sequential carburization strategy, in which a second carburization of the foam is performed at the surface of a β-SiC skeleton obtained during a first carburization step, as follows:

(1) First, a pre-shaped precursor polyurethane foam was impregnated/infiltrated with a phenolic resin containing micronized (<20 μm) metallic Si and carbon black powder.

(2) The shaped green body obtained was dried overnight at 120 °C and subsequently submitted to a first carburization reactive step under an Ar atmosphere for 1 h at 1360 °C. During the thermal treatment, the polyurethane was pyrolized giving almost no carbon yield, whereas the high carbon yield resin was pyrolized into amorphous carbon binding the carbon black and the Si powders together. Above 1000 °C, the carbon skeleton was subsequently attacked by SiO vapours formed by the reaction between Si and the residual traces of oxygen, whereas CO was allowed to carburize the metallic Si, yielding the formation of β-SiC according to eqn (1).

1 SiO_(g)_ + 2C_(s)_ → SiC_(s)_ + CO_(g)_

(3) The β-SiC skeleton foam was further impregnated by a Aeroxide® TiO_2_ P25 ethanolic suspension at 15 g L^−1^ by dipping the foam 50 times to get a 20 wt% TiO_2_ content (with intermediate rinsing steps with ethanol solution). The impregnated β-SiC skeleton foam was dried at 100 °C for 12 h. The impregnated β-SiC skeleton foam was dried at 100 °C for 12 h. The TiO_2_ wt% loading resulted from a preliminary study reported in ESI S1.†

(4) The foam was further submitted to a second reactive carburization step at 1360 °C for 1 h under an Ar atmosphere for forming a TiC shell using the residual unreacted carbon, according to eqn (2).

2 TiO_2(s)_ + 3C_(s)_ → TiC_(s)_ + 2CO_(g)_

(5) Finally, a thermal treatment in air was performed at temperatures between 550 °C and 700 °C for producing the final shell/core TiO_2_@β-SiC composite foams through the selective oxidation of the TiC skin into TiO_2_.

A reference β-SiC foam-supported TiO_2_ catalyst with 15 wt% of TiO_2_ was prepared *via* the classical deposition method consisting in successive dippings of the foam into a 15 g L^−1^ TiO_2_ P25 ethanolic suspension, with intermediate rinsing steps with ethanol solution, followed by drying at 100 °C and a final calcination treatment at 380 °C. Prior to deposition, the bare β-SiC foam was decarboned for 2 h at 700 °C in air for removing the residual unreacted carbon species by combustion. The TiO_2_ content of 15 wt% relative to the total foam weight was previously optimized^13^ and determined by weighing the TiO_2_/β-SiC foam.

### Evaluation of the photocatalytic efficiency

The experiments were carried out within a Suntest XLS+ reaction chamber (Atlas Material Testing Technology BV, Gelnhausen, Germany) equipped with a xenon arc lamp of 1700 W adjustable power and a Solar ID65 filter to limit the UV radiation at 300 nm for simulating solar exposition according to ICH Q1B guidelines. The runs were performed with simulated solar light at 250 W m^−2^ (300–800 nm), which corresponds to an average solar radiation in a summer's day in southern Europe (Fig. S2†). The reaction volume was 100 mL and the starting concentration of Diuron was 10 mg L^−1^. At each time interval, 5 mL of solution was sampled and then filtered through a 0.20 μm porosity filter to remove the photocatalyst powder if any, before the concentration of Diuron was determined by UV-visible spectrophotometry (Cary 100 scan Varian) by monitoring the disappearance of the main absorption peak at *λ* = 248 nm, and then total organic carbon (TOC) measurements were performed using a Shimadzu TOC-L analyzer to determine the organic carbon load.

### Stability test of the photocatalytic composite foams

The stability test protocol involved a two-step protocol consisting first in subjecting the foam to the reaction conditions and constraints of a photocatalytic test in pure distilled water. After 12 h under stirring with an applied irradiance of 60 W m^−2^, the foam was removed from the reactor before 4-chlorophenol, as a model substrate to degrade, was added at 20 mg L^−1^ to the solution potentially containing the TiO_2_ nanoparticles released from the foam. A photocatalytic test was subsequently performed following the same reaction conditions and protocol than in the case of the Diuron substrate.

### Characterisation techniques

X-ray diffraction (XRD) patterns were recorded on a D8 Advance Bruker diffractometer in a *θ*/*θ* mode, using the Kα1 radiation of a Cu anticathode (*λ* = 1.5406 Å).

The surface area measurements were carried out on a Micrometrics Tristar 3000 using N_2_ as the adsorbent at −196 °C with a prior outgassing at 200 °C overnight to desorb the impurities or moisture. The Brunauer–Emmett–Teller (BET) specific surface area was calculated from the N_2_ adsorption isotherm.

Scanning electron microscopy (SEM) was performed in secondary electron mode on a JEOL JSM-6700 F FEG microscope.

Thermogravimetric analysis (TGA) was carried out on a 20% (v/v) O_2_/N_2_ mixture at a 40 mL min^−1^ flow rate at a 10 °C min^−1^ heating rate in the 25–900 °C range with a Q5000TA analyzer.

X-ray photoelectron spectroscopy (XPS) characterization was performed on a Thermo VG Multilab ESCA3000 spectrometer (Al Kα anode at *hλ* = 1486.6 eV). The energy shift due to electrostatic charging was subtracted using the adventitious sp^2^ carbon C 1s band at 284.6 eV.

## Results and discussion

### Characterization of the TiO_2_@β-SiC composite foams

The main physico-chemical properties of the TiO_2_@β-SiC composite foams are shown in Table 1. The schematic diagram of the sequential two-step carburization SMS replica synthesis shows optical images of the pre-shaped alveolar polyurethane precursor and of the composite foam at different steps of the replica synthesis process (Fig. 1). This evidenced that the original macrostructural features of the alveolar foams were retained throughout the multi-step sequential carburization SMS replica process, going from the pre-shaped polyurethane foam to the ready-to-use TiO_2_@β-SiC composite foam (Table 1). Notably, both the alveolar open-cell structure and the cell size of the foam were maintained during each carburization step and the final calcination treatment.

**Table 1:** Main physico-chemical properties of the TiO_2_@β-SiC composite foams

	β-SiC	TiO_2_@β-SiC-550	TiO_2_@β-SiC-600	TiO_2_@β-SiC-700
TiO_2_ crystallized phases (anatase/rutile)	—	97 : 3	65 : 35	63 : 37
TiO_2_ mean crystallite size (anatase/rutile) (nm)^a^	—	11/—	12/10	12/11
*S*_BET_ surface area (m^2^ g^−1^)	25	48	38	35
Residual carbon content (wt%)^b^	0	4	0.5	0
Mean cell size (*φ*) (μm)	5400 ± 700	5000 ± 600	5100 ± 500	5200 ± 400
Window size (*a*) (μm)	2300 ± 575	2300 ± 300	2500 ± 500	2400 ± 400
Bridge diameter (*d*_s_) (μm)	575 ± 80	510 ± 30	545 ± 50	530 ± 20
Open porosity^c^	0.91 ± 0.05	0.94 ± 0.05	0.94 ± 0.05	0.94 ± 0.05
Geometrical specific surface area (m^−1^)^d^	620 ± 200	460 ± 200	440 ± 200	453 ± 200

a Determined from the XRD analysis by applying the Scherrer equation to the (101) and (110) peaks of anatase and rutile, respectively, with the classical assumption of spherical crystallites.

b The residual unreacted carbon content was determined by TGA analysis by evaluating the weight loss observed between 500 °C and 800 °C.

c Measured by water displacement.

d Derived from the model developed by Edouard and colleagues using the open porosity and the mean strut diameter.^33,34^

Similar to the reference β-SiC foams, the TiO_2_@β-SiC composite foams could be manufactured with adjustable shapes suitable for adaption to the reactor geometry, and they were prepared as 5 cm (*d*) × 2 cm (*h*) disks. The TiO_2_@β-SiC composite foams and the reference β-SiC foam displayed globally similar macroscopic features in terms of their characteristic parameters, with a cell size of 5200 ± 400 μm, a window size of 2380 ± 400 μm and a strut diameter at 540 ± 50 μm, so that they exhibited a similar open porosity of *ca.* 0.91–0.94, and a similar geometrical surface area in the 440–620 m^−1^ range. One can thus stress that the reference TiO_2_/β-SiC foam and the newly developed TiO_2_@β-SiC composite foams showed similar light transmission profiles through the foams.^7^

**Fig. 2:**
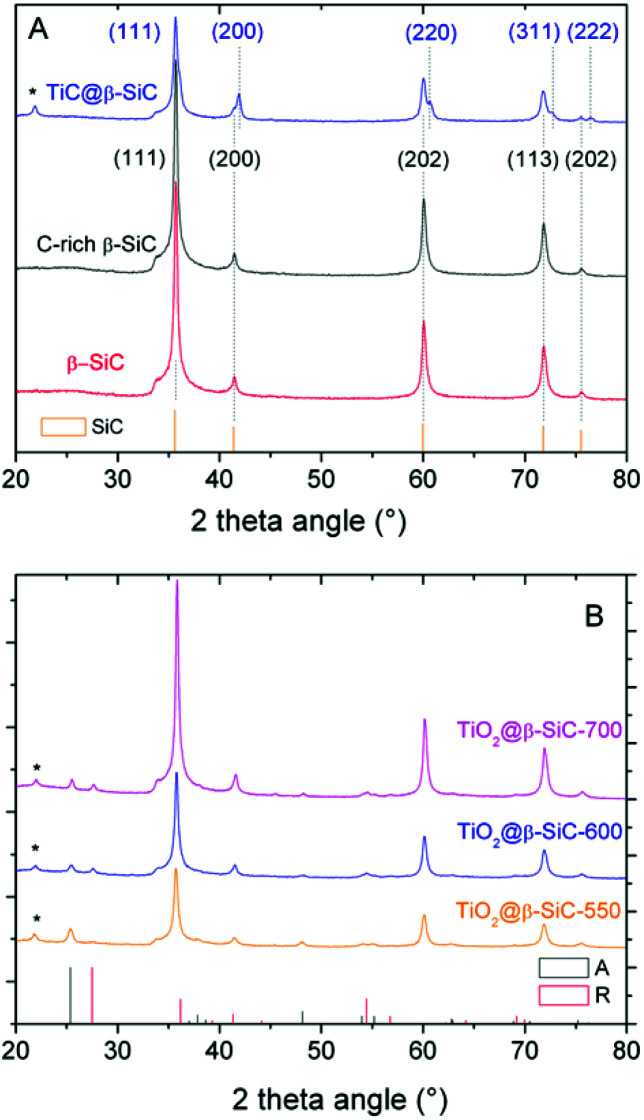
XRD patterns of the β-SiC-based foams at different steps of the synthesis protocol: (A) the bare alveolar β-SiC foam obtained through the classical SMS replica method, the C-rich β-SiC foam after the 1st carburization step and the shell/core TiC–β-SiC composite foam after the 2nd carburization step; (B) the TiO_2_–β-SiC composite foams after calcination at 550 °C, 600 °C and 700 °C. (*) Most intense diffraction peak of crystallized SiO_2_ (JCPDS card 65-0466).

The XRD patterns of the composite foams at different steps of the replica SMS are shown in Fig. 2A and B. They all exhibited diffraction lines corresponding to the (111), (200), (202), (311) and (222) planes of the β-SiC polymorph in the fcc structure,^23^ which were similar to those of both the reference β-SiC foam and the β-SiC foam after the first carburization steps (labelled as C-rich β-SiC foam considering the presence of residual unreacted carbon species, see later Fig. 5A). After the 2nd carburization step at 1360 °C, the pattern revealed the TiC@β-SiC nature of the composite foam, with the diffraction lines corresponding to the (111), (200), (220), (311) and (222) planes of TiC in a fcc structure.^16,24^ The TiC@β-SiC composite foam contained as well a small amount of crystallized SiO_2_, as evidenced by the presence of an additional peak at 22.0° characteristic of the diffraction of the (101) planes of crystallized SiO_2_. This could result from the inhomogeneity of the carbon distribution within the shaped infiltrated body – due to the inhomogeneity either of the foam coating or within the infiltration slurry – which could lead to the existence of carbon-poor zones during the foam carburization, and therefore to local deficits in reductive carbon compared to the silicon species. The TiC@β-SiC composite foam can be considered as a shell/core foam, since the 2nd carburization reaction occurred between the unreacted carbon species of the C-rich β-SiC foam and the TiO_2_ exclusively located at the skeleton foam surface.

The XRD patterns of the composite foams after calcination shown in Fig. 2B evidenced the selective oxidation of the TiC phase into TiO_2_ – thanks to the β-SiC resistance to oxidation^25^ – with the characteristic most intense peaks at 25.3° and 27.1° corresponding to the diffraction of the (101) and (110) planes of anatase (JCPDS card 21-1272) and rutile (JCPDS card 21-1276), respectively. At 550 °C, the TiO_2_@β-SiC composite foams had an anatase : rutile ratio of 97 : 3, which decreased to 65 : 35 and 63 : 37 at 600 °C and 700 °C, respectively. Although the rutile content increased with increasing the calcination temperature, it remained lower than that of TiO_2_–β-SiC composite powders, which increased up to 70% when calcined at 600 °C.^16^ Indeed, whereas β-SiC can act as a thermal regulator to disperse the heat issued from the TiC oxidation thanks to its high thermal conductivity,^25^ the continuous aspect of the self-bonded (*i.e.* binder-less) foams is known to improve the heat transfer through the whole matrix.^26^ This explained as well that the calcination temperature did not impact on the TiO_2_ mean crystallite size, with a mean size of around 11 nm.

The TiO_2_@β-SiC composite foams had a higher surface area within the 35–48 m^2^ g^−1^ range when compared to that of the reference β-SiC foam at 25 m^2^ g^−1^. This probably resulted from the presence in the foams of residual unreacted carbon with adsorption properties. Indeed, increasing the calcination temperature from 550 °C to 700 °C caused a decrease in the surface area of the composites, attributed to the combustion of the residual carbon. At 700 °C, the surface area might be explained by the possible formation of voids and cracks during the TiC to TiO_2_ oxidation, as reported in the case of TiO_2_–β-SiC composite powder systems.^16^

**Fig. 3:**
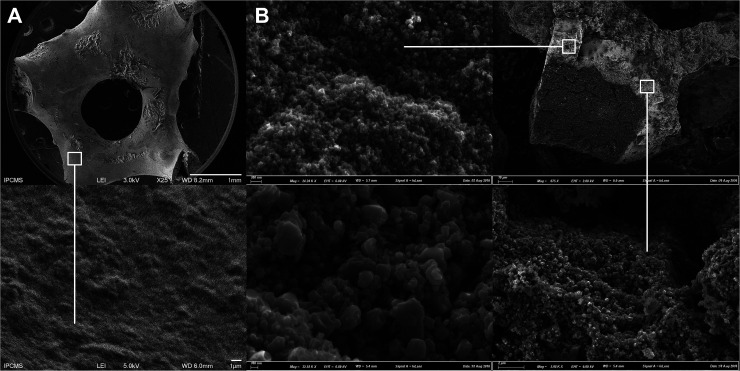
SEM images of the: (A) the bare β-SiC foam and (B) the TiO_2_@β-SiC composite foam after calcination at 550 °C. Cross-section image of a broken foam bridge, evidencing the TiO_2_ skin and the β-SiC core.

The heterogeneous nature of the TiO_2_@β-SiC composite foam surface compared to the surface of the bare β-SiC foam was evidenced in the SEM images of Fig. 3. Further, the shell/core nature of the TiO_2_@β-SiC composite foams was confirmed in the cross-section SEM images of a broken foam bridge, showing the existence of a thin skin located at the surface of a β-SiC core. EDS analysis carried out on the foam core revealed the absence of any titanium atoms, confirming its pure β-SiC nature, whereas both titanium and silicon elements were observed during the EDS analysis of the external skin, due to the analysis depth in SEM (not shown).

**Fig. 4:**
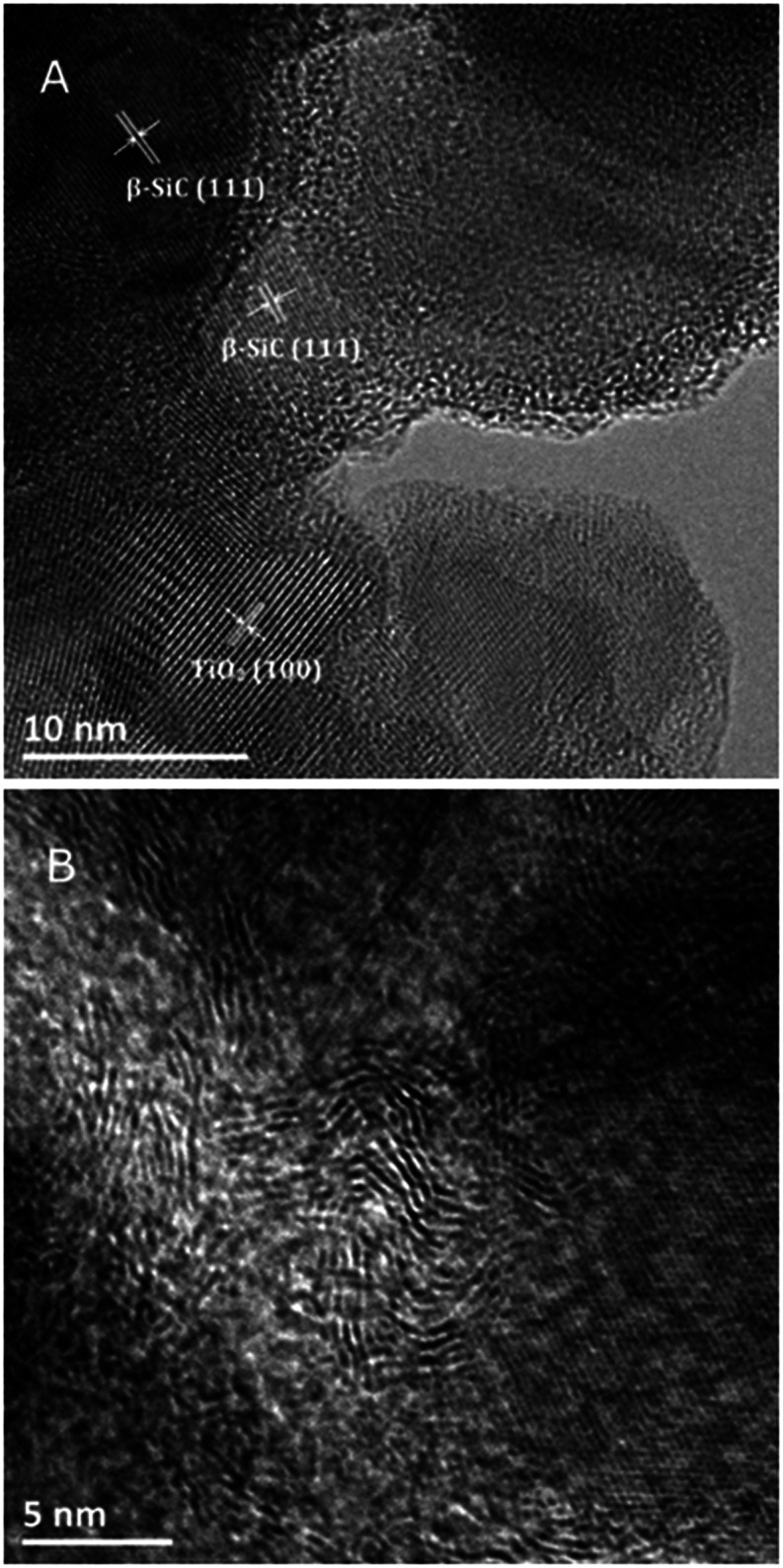
(A and B) TEM images of the TiO_2_@β-SiC composite foams calcined at 550 °C. The (111) and (101) planes of β-SiC and anatase TiO_2_ have interplanar distances of 2.5 Å (JCPDS no. 00-029-1129) and 3.5 Å (JCPDS no. 00-021-1272), respectively.

The TEM images of the TiO_2_@β-SiC composite foam calcined at 550 °C shown in Fig. 4A evidenced the proximity of both β-SiC and anatase TiO_2_ crystallites in the photocatalyst, with interplanar spacings of 2.5 Å and 3.5 Å, consistent with the (111) and (101) planes of β-SiC and anatase TiO_2_ phases, respectively. In addition, Fig. 4B shows the residual presence of turbostratic carbon in the sample calcined at 550 °C.

### Dark adsorption experiments

**Fig. 5:**
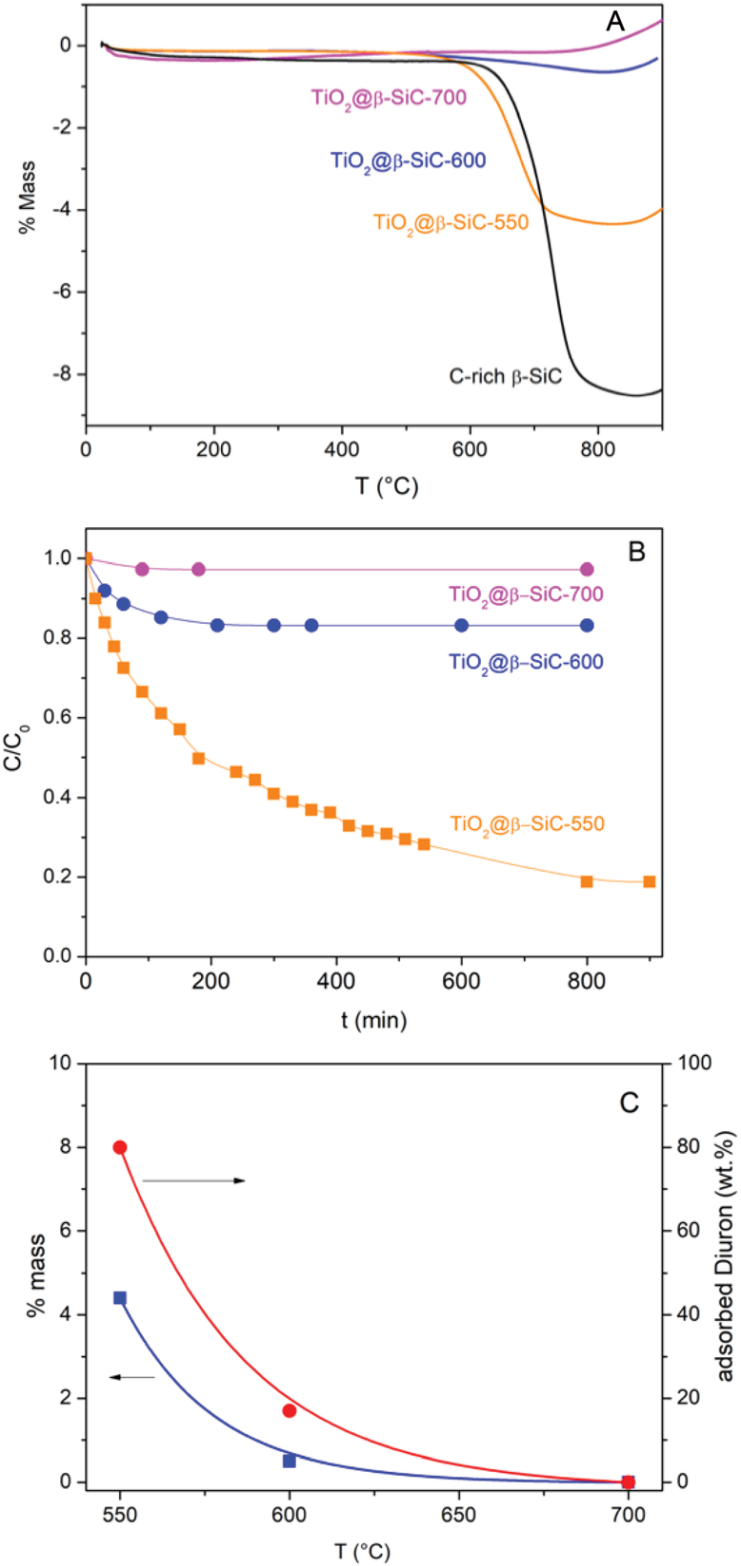
(A) TGA profiles of the C-rich β-SiC skeleton foam after the 1st carburization step and of the TiO_2_@β-SiC composite foams after calcination; (B) dark adsorption experiments performed on the TiO_2_@β-SiC composite foams; (C) influence of the calcination temperature of the composite foams on both the wt% of residual carbon and the wt% of Diuron adsorption.

The amount of residual unreacted carbon in the carbon-rich β-SiC skeleton foam after the 1st carburization step and in the TiO_2_@β-SiC composite foams after calcination was determined by TGA experiments (Fig. 5A). The TGA profiles showed that the carbon-rich β-SiC skeleton foam contained 8 wt% of excess carbon, whereas the calcination applied to the TiC@β-SiC composite after the 2nd carburization of the foam for obtaining TiO_2_ from TiC directly influenced the amount of residual unreacted carbon remaining within the TiO_2_@β-SiC foams. A final calcination in air at 700 °C was necessary for completely removing the residual carbon by combustion, whereas the TiO_2_@β-SiC composite foams calcined at 550 °C and 600 °C contained about 4 wt% and 0.5 wt% of unreacted carbon (Table 1). The small weight gain above 800 °C corresponded to the high temperature surface oxidation of SiC into SiO_2_ as usually observed for SiC prepared *via* the SMS replica method.

The evolution of the Diuron concentration during the dark adsorption period on the TiO_2_@β-SiC composite foams calcined at temperatures in the 550–700 °C range is shown in Fig. 5B for a 10 mg L^−1^ initial Diuron concentration and in Fig. S4† for a large range of concentrations (5, 10 and 20 mg L^−1^). The adsorption properties of the composite foams were strongly influenced by the final calcination temperature, in agreement with the TGA profiles. Indeed, no significant adsorption of Diuron was obtained on the composite foams calcined at 700 °C, while about 20% and 80% adsorption of Diuron was achieved on the composite foams calcined at 550 °C and 600 °C for a 10 mg L^−1^ initial Diuron concentration.

Further, the influence of the amount of residual unreacted carbon in the composite on the adsorption profile was evidenced independently of the initial Diuron concentration (Fig. S4†). The higher the amount of residual unreacted carbon in the composite foam, the higher the specific surface area developed by the foam and consequently the more pronounced the adsorption profile, and the higher the Diuron amount removed by adsorption whatever the initial concentration.

So, the selection of the final calcination temperature (550–700 °C range) appeared as an easy way to vary the amount of residual un-combusted carbon from 0 to 4 wt%, and consequently, to tune the adsorption properties of the TiO_2_@β-SiC composite foams, as materialized in Fig. 5C.

### Photocatalytic runs

**Fig. 6:**
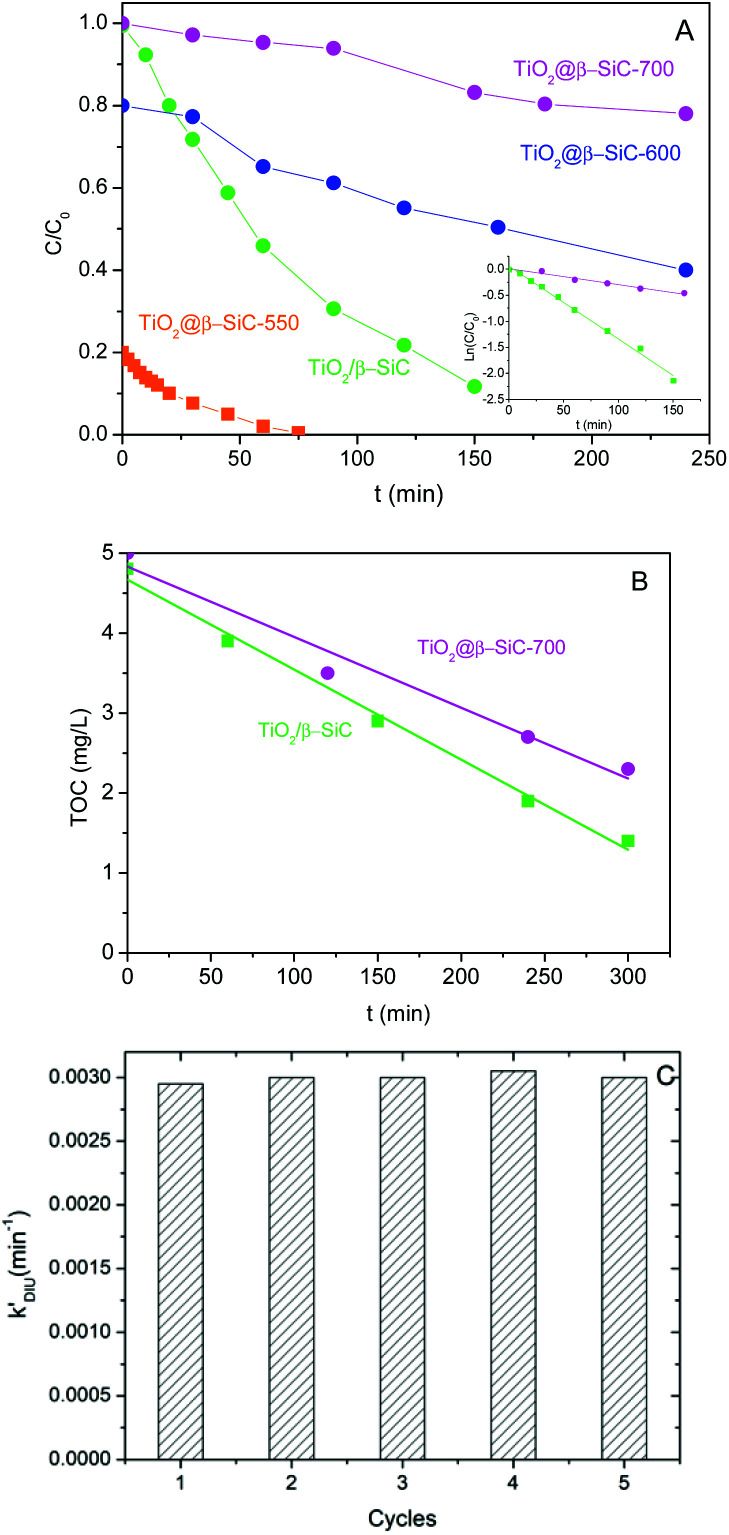
(A) Diuron photodegradation kinetics and (B) mineralization kinetics obtained on the TiO_2_@β-SiC composite foams. Comparison with a reference TiO_2_ (15 wt%)/β-SiC foam photocatalyst; (C) Diuron photodegradation kinetics observed on the TiO_2_@β-SiC-700 composite foam with consecutive runs.

Fig. 6A shows that the TiO_2_@β-SiC composite foams were active for degrading Diuron under solar light, whatever their adsorption properties, *i.e.* whatever the remaining Diuron concentration to be removed after the adsorption step – 10, 8 or 2 mg L^−1^ for foams calcined at 700 °C, 600 °C and 550 °C, respectively. The higher the adsorption capacity, the faster the reduction of the Diuron concentration in water. However, the kinetic rate constants were not compared to that achieved on the TiO_2_@β-SiC composite foam calcined at 700 °C, due to the far different initial concentrations of Diuron to be degraded. Calcined at 700 °C, the composite foam exhibited a lower activity in terms of Diuron degradation than the reference TiO_2_/β-SiC foam prepared *via* the impregnation and stabilization of TiO_2_ on a conventional β-SiC foam, with an apparent kinetic constant of 3 × 10^−3^ min^−1^*vs.* 14 × 10^−3^ min^−1^, respectively.

In contrast, the superiority of the reference TiO_2_/β-SiC foam over the TiO_2_@β-SiC composite foam was less pronounced in terms of TOC removal, with a kinetic constant of 12 × 10^−3^ mg L^−1^ min^−1^*vs.* 9 × 10^−3^ mg L^−1^ min^−1^, respectively (Fig. 6B). This behaviour might result from an enhanced degradation of the reaction intermediates on the composite foam compared to the reference foam counterpart.

Several groups evidenced that the Diuron degradation pathways were not affected when TiO_2_ was associated with fibres and SiO_2_ binder, or to Pt.^19,27^ Matos *et al.* showed that associating TiO_2_ with carboned adsorbents did not modify the reaction mechanism and similar reaction intermediates were observed using 4-chlorophenol, phenol and 2,4-dichlorophenoxyacetic acid as model pollutants to degrade, compared to bare TiO_2_ (even if the product distribution might differ in some cases).^28,29^ We thus proposed that the mechanism for the Diuron photocatalytic degradation was not significantly influenced by the adsorption properties of the TiO_2_@β-SiC composite foams.

**Fig. 7:**
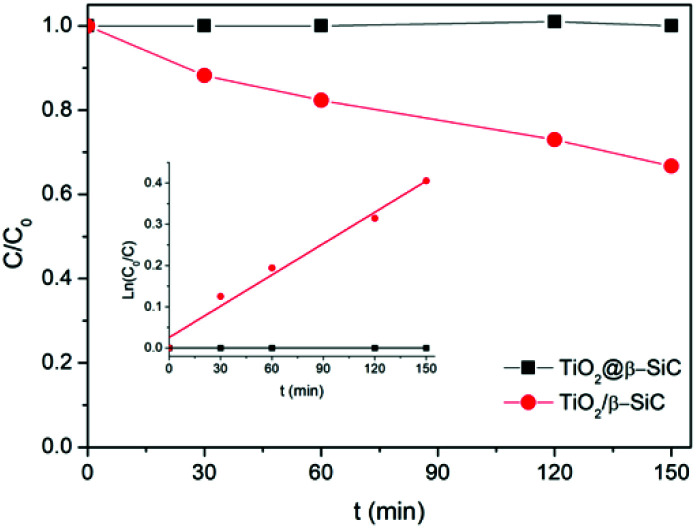
4-Chlorophenol photocatalytic degradation kinetics obtained during the stability test. Reaction conditions: [4-CP]_0_ = 20 mg L^−1^; *T* = 25 °C; UV-A irradiance of 60 W m^−2^.

Finally, the stability of the TiO_2_@β-SiC-700 composite foam was evaluated by studying its ability to be reused, as well as by submitting the foam to a stability test procedure. First, the photocatalytic foam displayed good reusability when performing several sequential runs, with apparent kinetic rate constants for Diuron degradation calculated on average as 2.9 × 10^−3^ min^−1^ ± 0.1 × 10^−3^ min^−1^, respectively (Fig. 6C). Further, the implementation of the stability test procedure evidenced the superior stability of the TiO_2_@β-SiC composite foam when compared to the TiO_2_-supported β-SiC foam counterpart (Fig. 7). Indeed, after the first stirring treatment with the composite foams in pure water under UV-A, no 4-chlorophenol degradation was observed, whereas a degradation of 30% was obtained after a 150 min test in the case of the TiO_2_/β-SiC foam. Although it cannot be ruled out that the TiO_2_ nanoparticles potentially released from the composite foam displayed a lower photocatalytic activity than the TiO_2_ P25 released from its supported counterpart, the amount of TiO_2_ released from the supported photocatalyst was estimated at 0.06 g L^−1^ using a calibration curve (Fig. S3†), while in contrast, it remained lower than 0.001 g L^−1^ in the case of the composite foam.

**Fig. 8:**
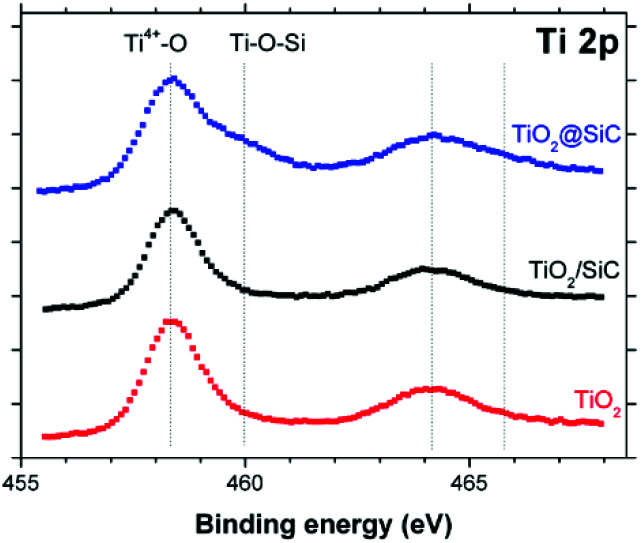
Ti 2p orbital XPS spectra recorded on the TiO_2_@β-SiC composite foams, in comparison to the β-SiC-supported TiO_2_ counterpart and a reference TiO_2_ powder (Aeroxide© P25 from Evonik).

It is proposed that the superior stability of the TiO_2_@β-SiC composite foam resulted from the existence of Ti–O–Si cross-linking bonds between the external TiO_2_ shell and the surface of the β-SiC skeleton core. Indeed, while the Ti_2p_ orbital XPS spectra of the composite foam showed the typical Ti_2p_3/2__–Ti_2p_1/2__ doublet located at 458.3 and 464.0 eV with a spin–orbit splitting of 5.7 eV, ascribed to Ti^4+^ (Ti–O) surface species in TiO_2_ observed in the case of bulk TiO_2_ powder and of the β-SiC foam-supported TiO_2_ counterpart, a higher energy contribution was observed and assigned to Ti–O–Si bonds, in agreement with the literature (Fig. 8).^30–32^

## Conclusions

Coating-free TiO_2_@β-SiC photocatalytic composite foams were synthesized through a sequential multi-step SMS replica method, in which an external TiO_2_ layer was formed by the selective oxidation of an external TiC skin obtained by carburization of a TiO_2_ coating at the surface of a β-SiC skeleton foam synthesized from a pre-shaped polymer foam during a first carburization step. These ready-to-use shell/core alveolar media exposed as an irradiated surface the TiO_2_ photocatalyst, while the macroscopic structure was provided by the β-SiC alveolar foam, so that no post-synthesis immobilization or synthesis process of the active phase onto the foam support was necessary. Further, applying a final calcination treatment to the carbide foams (550–700 °C) allowed the amount of residual unreacted carbon within the active shell/core TiO_2_@β-SiC composite foams to be tuned, and consequently their adsorption behaviour towards the Diuron pollutant in water to be tuned. The lower the calcination temperature, the more pronounced the adsorption profile of the composites and the higher the Diuron amount removed by adsorption on the residual unreacted carbon. The ready-to-use TiO_2_@β-SiC composite foams were active in the degradation of the Diuron pesticide in water, although remaining less efficient than the reference β-SiC-supported foam TiO_2_ photocatalyst counterpart. However, they displayed a good reusability with test cycles and benefitted from an enhanced stability in terms of titania release to water.

## Conflicts of interest

There are no conflicts to declare.

## Supplementary Material

RA-010-C9RA09553E-s001
