# Supplementary material for: Coating-free TiO2@β-SiC alveolar foams as a ready-to-use composite photocatalyst with tunable adsorption properties for water treatment
Source: RSC Adv. 2020 Jan 22;10(7):3817–25. doi: 10.1039/c9ra09553e (PMC9048602; doi:10.1039/c9ra09553e)
Supplement: RA-010-C9RA09553E-s001 [file RA-010-C9RA09553E-s001.pdf]

Electronic Supplementary Material (ESI) for RSC Advances.
This journal is © The Royal Society of Chemistry 2020

Supplementary material

Coating-free TiO_2@β-SiC alveolar foams as ready-to-use composite photocatalyst with tunable adsorption properties for water treatment

Marisa Rico-Santacruz[1], Patricia García-Muñoz [1]*, Clément Marchal[1], Nelly Batail[2], Charlotte Pham[2], Didier Robert[1], Nicolas Keller[1]*

(1) Institut de Chimie et Procédés pour l'Energie, l'Environnement et la Santé (ICPEES), CNRS/University of Strasbourg,25 rue Becquerel 67087 Strasbourg, France.

(2) SICAT SARL, 20 Place des Halles,67000 Strasbourg, France.

Corresponding authors(*):garciamunoz@unistra.fr;nkeller@unistra.fr

Figure S1. **(A)** Dark adsorption experiments performed on the TiO_2@β-SiC composite foams with different TiO_2 wt.% contents and calcined at 600°C ; **(B)** Diuron photodegradation kinetics obtained on those TiO_2@β-SiC composite foams, with the corresponding apparent kinetic rate constant (pseudo first order reaction rate).

Figure S2. Spectral distribution of the simulated solar light.

Figure S3. Calibration curve expressing the influence of the TiO_2 concentration on the apparent rate constant for the chlorophenol degradation (pseudo first order reaction). $[4\text{-CP}]_0$ = 20 mg/L; T=25°C; UV-A irradiance of 60 W m^{-2} .

Figure S4. **A)** Dark adsorption experiments performed on TiO_2@β-SiC composite foams varying in terms of residual carbon content for different initial Diuron concentrations. **B)** Amount of diuron adsorbed as a function of the residual carbon content in wt.% for different initial Diuron concentrations.

Figure S1

For a preliminary study, TiO_2@β-SiC composite foams were prepared with a TiO_2 content of 11 wt.%, 20 wt.% and 33 wt.% on the β-SiC skeleton foam during the step 3 of the sequential multi-step carburization SMS method. The TiO_2@β-SiC composite foams obtained after final calcination at 600°C displayed similar Diuron adsorption behavior in the dark indepently of the TiO_2 content, as depicted in Fig. S1A. By contrast, Fig. S1B evidenced the superiority of the composite foam with a 20 wt.% TiO_2 content in terms of Diuron removal, as evidenced by the higher apparent kinetic rate constant for Diuron degradation, calculated at 3.1 10^{-3} min^{-1}. Therefore, the main part of the work was further performed on composite foams obtained by impregnating the β-SiC skeleton foam with TiO_2 at a 20 wt.% content.

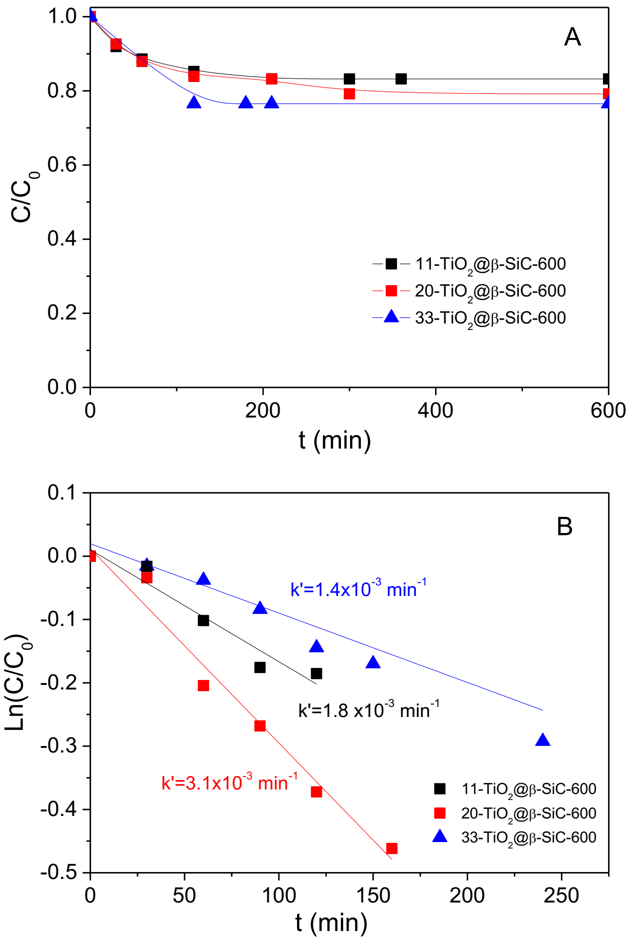

Figure S1 (A) Dark adsorption experiments performed on the TiO_2@β-SiC composite foams with different TiO_2 wt.% contents and calcined at 600°C ; **(B)** Diuron photodegradation kinetics obtained on those TiO_2@β-SiC composite foams, with the corresponding apparent kinetic rate constant (pseudo first order reaction rate).

Figure S2

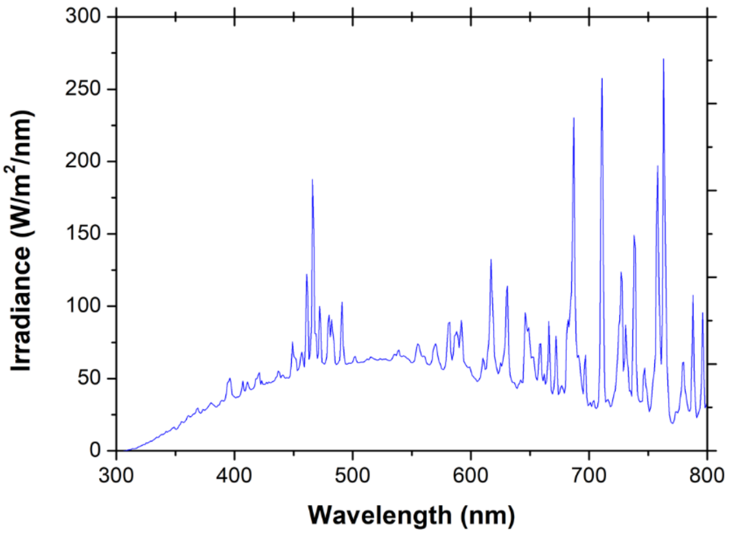

Figure S2. Spectral distribution of the simulated solar light.

Measurements have been performed using a wideband RPS900-W rapid portable spectroradiometer from International Light Technology.

Figure S3

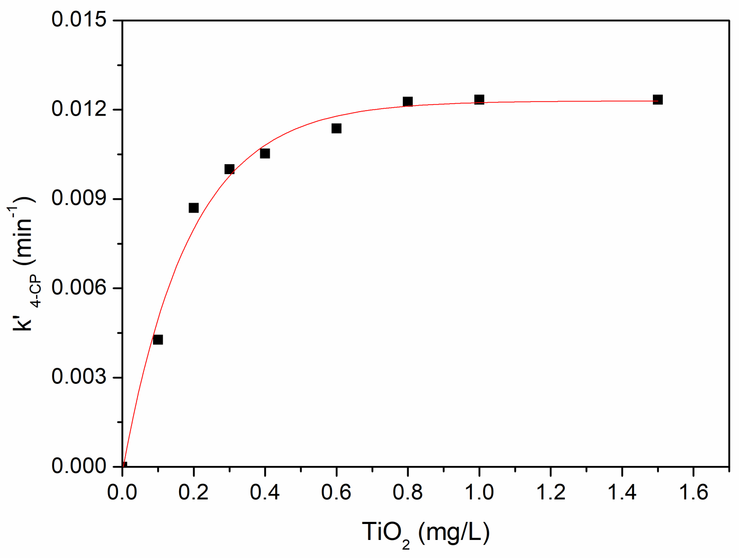

Figure S3. Calibration curve expressing the influence of the TiO_2 concentration on the apparent rate constant for the chlorophenol degradation (pseudo first order reaction). $[4\text{-}CP]_0$ = 20 mg/L; T=25°C; UV-A irradiance of 60 W m^{-2}.

Figure S4

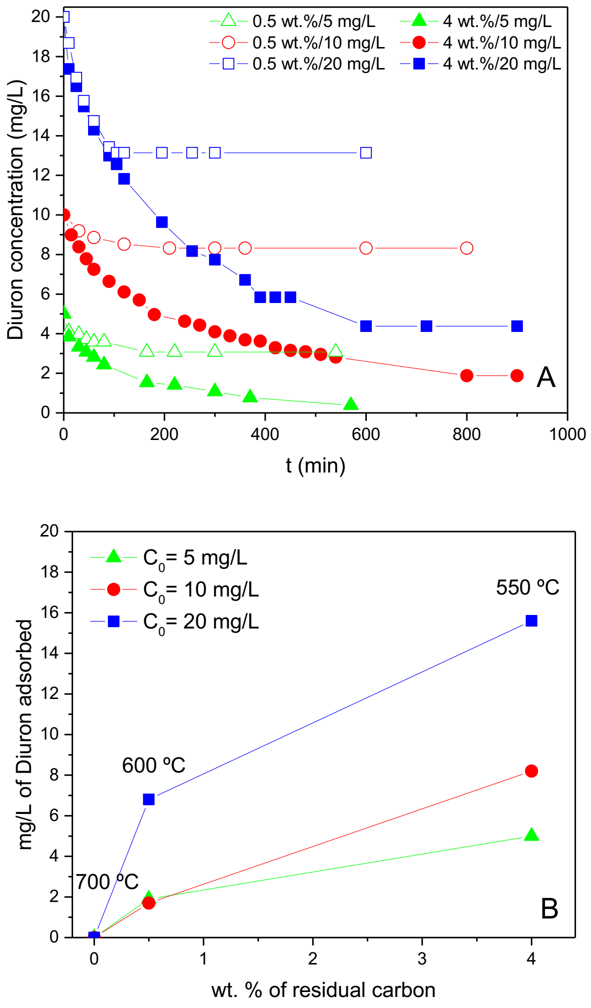

Figure S4. A) Dark adsorption experiments performed on TiO_2@β-SiC composite foams varying in terms of residual carbon content for different initial Diuron concentrations. **B)** Amount of Diuron adsorbed as a function of the residual carbon content in wt.% for different initial Diuron concentrations.
